# ‘Changing Minds’: determining the effectiveness and key ingredients of an educational intervention to enhance healthcare professionals’ intentions to prescribe physical activity to patients with physical disabilities

**DOI:** 10.1186/1748-5908-9-30

**Published:** 2014-03-01

**Authors:** Jennifer R Tomasone, Kathleen A Martin Ginis, Paul A Estabrooks, Laura Domenicucci

**Affiliations:** 1 Department of Kinesiology, McMaster University, Ivor Wynne Centre E110, 1280 Main Street West, Hamilton, Ontario L8S 4K1, Canada; 2 Virginia Tech and Carilion Clinic, 1 Riverside Circle, SW Roanoke, Virginia, USA; 3 The Canadian Paralympic Committee, 225 Metcalfe Street, Suite 310, Ottawa, Ontario, Canada

**Keywords:** Educational intervention, Healthcare professionals, Leisure-time physical activity, Physical activity prescription, Physical disability, Theory of Planned Behaviour

## Abstract

**Background:**

Healthcare professionals (HCPs) are vital conduits of leisure-time physical activity (LTPA) information; however, few discuss LTPA with their patients with disabilities. ‘Changing Minds, Changing Lives’ (CMCL) is a nationwide, theory- and evidence-based seminar aimed at increasing LTPA-discussion among HCPs by enhancing their attitudes, subjective norms, perceived behavioural control (PBC), and intentions. The purposes of the current study were to: examine the effectiveness and short- and long-term maintenance of a CMCL seminar on HCPs’ social cognitions to discuss LTPA; and explore key implementation variables that predict changes in HCPs’ social cognitions.

**Methods:**

Prior-to, as well as immediately, one, and six months following a CMCL seminar, 97 HCPs (M_age_ ± SD = 36.23 ± 10.42; 69.0% female; 97.9% Caucasian; 38.1% rehabilitation therapists; years in profession = 11.56 ± 9.94) from five Canadian provinces completed questionnaires that assessed the Theory of Planned Behaviour constructs with regard to discussing LTPA with their patients with a physical disability. Key presenter characteristics and intervention delivery components were extracted from presenter demographic questionnaires and seminar checklists, respectively. Separate repeated-measures ANOVAs and post-hoc t-tests evaluated changes in HCPs’ social cognitions. Hierarchical multiple regressions were conducted to predict intentions and to understand which implementation variables may help explain significant changes in social cognitions.

**Results:**

Significant increases in HCPs’ social cognitions for discussing LTPA were reported from pre- to post-seminar (ps <0.002); however, increases were not maintained at follow-up. PBC emerged as the strongest predictor of participants’ post-CMCL intentions (β = 0.45, p <0.001). Although several implementation characteristics were related to changes in perceptions, the number of seminars the presenter delivered was the only significant negative predictor of post-seminar PBC (β = -0.18, p <0.05).

**Conclusions:**

Future iterations of the CMCL intervention should include additional strategies to sustain improvements in HCPs’ social cognitions over time. Future CMCL evaluations should measure additional implementation variables so that the key ingredients for ‘Changing Minds’ can continue to be investigated.

## Background

Among individuals with a physical disability, participation in leisure-time physical activity (LTPA) has been shown to be associated with numerous physical, psychological, social, and quality of life benefits [1-5]. However, only 3% of the 4.4 million Canadians with a physical disability engage in LTPA [6,7]. Despite recent efforts to develop informational resources to increase LTPA participation, such as physical activity guidelines [8,9], adults with a physical disability remain one of the most inactive segments of the population [6], in part due to a perception of a lack of accessible LTPA information [10]. The delivery of LTPA information is an essential component of strategies aimed at increasing LTPA participation in the disability community.

Individuals with physical disabilities have identified healthcare professionals (HCPs) – including physicians, nurses, rehabilitation therapists, and kinesiologists – as desired and credible messengers for delivering LTPA information [11,12]. However, very few HCPs discuss and prescribe LTPA [13] as they often lack the knowledge, confidence, and resources to do so [14,15]. To address these barriers, the Canadian Paralympic Committee offers ‘Changing Minds, Changing Lives’ (CMCL), a nationwide, seminar-mediated educational intervention designed to provide HCPs with the knowledge, strategies and resources needed to discuss and prescribe LTPA to their patients with physical disabilities [16]. Briefly, CMCL seminars are designed to be a single, hour-long session delivered by a HCP and a physically active individual with a physical disability. The seminar curriculum is embedded within a PowerPoint presentation and includes up-to-date research evidence and Canadian statistics regarding LTPA participation among both the able-bodied and physically disabled populations, information demonstrating that HCPs are key influencers in the lives of their patients and are expected to play a role in promoting LTPA, and strategies that HCPs can use for discussing LTPA (*e.g*., asking a few key questions during routine patient appointments and providing referrals to, and information on, LTPA resources). CMCL encourages a variety of types of LTPA, including structured exercise, sport activities, and active play. Parasport – parallel sport opportunities for people with physical disabilities (*e.g*., wheelchair basketball, sit-skiing) – is a particular focus of CMCL because the Canadian Paralympic Committee is dedicated to inspiring all Canadians with disabilities to get involved in sport. Presenters are encouraged to use the set curriculum and to adapt other seminar components for the local presentation context and resources available to them (*e.g*., educational handouts about local LTPA options for people with a physical disability, and show-and-tell of adaptive LTPA equipment). (For more details about the content of the CMCL seminars and how presenters are trained, please refer to references [16]; Tomasone, Martin Ginis, Estabrooks & Domenicucci: ‘Changing Minds, Changing Lives’ from the top-down: An investigation of the dissemination and adoption of a nationwide educational intervention to enhance health care professionals’ intentions to prescribe physical activity, resubmitted]).

The CMCL curriculum used across Canada is evidence-based and founded in the Theory of Planned Behaviour (TPB) [Tomasone et al., resubmitted]. According to the TPB, an individual’s intentions (or motivation) to perform a target behaviour (*e.g*., discussing and prescribing LTPA) is influenced by his/her attitudes, subjective norms, and perceived behavioural control (PBC) for the behaviour [17]. In turn, intentions and PBC are direct predictors of behaviour. The TPB has been suggested to be an ideal behaviour change framework for understanding and promoting knowledge mobilization among HCPs [18-20], as it accounts for a variety of factors known to influence professional behaviour across different HCP populations, behaviours and contexts. By targeting the TPB social cognitions (*e.g*., attitudes, subjective norms, and PBC) to discuss and prescribe LTPA through an educational intervention like CMCL, it may be possible to alter HCPs’ LTPA prescription intentions. Theory has not been used extensively in implementation research. A recent systematic review of theory use in implementation studies (including educational interventions) found that only 22.5% of studies used theory; furthermore, only 6% of studies explicitly designed the intervention and tested hypotheses based on theory [21]. The present study makes a significant contribution to implementation research by using theory (TPB) to guide the development of the CMCL intervention and to develop and test hypotheses to evaluate the intervention’s effectiveness.

However, the challenge of implementing educational interventions, such as CMCL, in real-world practice is that implementation varies over time and across providers [22,23]. Identifying the key components associated with a greater likelihood of success in changing HCP cognitions and behaviour would assist with the development of more potent and cost-effective interventions [23,24], ultimately moving the science and practice of implementing HCP behaviour change interventions forward. Using a theory, such as the TPB, to hone in on the critical modifiable and non-modifiable intervention components that predict intervention effectiveness will extend previous work examining the relationship between implementation and effectiveness [23], as well as help isolate predictors of HCP behaviour change [25].

As such, the first purpose of the current study was to examine the effectiveness and short- and long-term maintenance of a theory- and evidence-based intervention on HCPs’ social cognitions for discussing and prescribing LTPA to their patients with a physical disability. We hypothesized that HCPs would report significant increases in attitudes, subjective norms, PBC, and intentions to discuss LTPA with their patients with physical disabilities immediately following their attendance at a CMCL seminar. If significant increases in cognitions were seen, we also wanted to explore whether the TPB constructs could predict HCPs’ intentions to discuss LTPA. As the TPB has been shown to predict HCPs’ clinical behaviours other than LTPA prescription [18-20], we hypothesized that attitudes, subjective norms, and PBC would emerge as significant predictors of intentions. Given that CMCL is a single, seminar-mediated intervention without follow-up strategies known to enhance long-term behaviour change [26], we hypothesized that changes in HCPs’ social cognitions would not be maintained at 1- and 6-month follow-up.

A secondary purpose was to explore the key implementation variables that predict changes in HCPs’ social cognitions. In line with Durlak and DuPre’s ecological framework for understanding effective implementation [23], we considered both presenter characteristics and intervention delivery components that may influence intervention effectiveness. Given the CMCL presenters’ role in persuading attendees to discuss LTPA, we hypothesized that presenter characteristics would predict changes in HCPs’ subjective norms. Given that CMCL was designed to relay information, resources and strategies for discussing LTPA with patients, we also hypothesized that intervention delivery components would predict changes in HCPs’ attitudes and PBC for discussing LTPA with their patients with physical disabilities.

## Methods

### Participants

This process evaluation study was conducted within the existing delivery of the CMCL intervention program in Canada, with the research team and CMCL staff working together to implement the evaluation measures alongside CMCL’s standard protocol. In line with existing delivery protocol, CMCL Provincial Coordinators from Canadian provinces contacted and organized seminars for interested healthcare institutions (*e.g*., community care access clinics, hospitals) in their province, and healthcare institutions invited their staff to participate in the seminars. Additional recruitment for the study outside the standard CMCL protocol was not conducted by the research team. The costs associated with the delivery of the CMCL intervention were covered by the Canadian Paralympic Committee. A total of 15 CMCL seminars were delivered to 324 HCPs across Canada during the study period (November 2011 to August 2012). Upon arrival at the seminar, each HCP received a copy of the letter of information/consent. Of the 324 attendees, 97 HCPs consented to participate in the current study (29.9% participation rate). The majority of participants were female (67.0%) and Caucasian (97.9%). A large percentage of participants were from New Brunswick (32.0%) and worked as rehabilitation therapists (*e.g*., physical therapists, occupational therapists, recreational therapists; 38.1%). Participants’ average age was 36.23 years (SD = 10.42) and had worked in their careers as HCPs for a mean of 11.56 years (SD = 9.94). Participants engaged in LTPA regularly (M ± SD = 4.35 ± 1.71 days/week); however, only 3.1% of participants were involved in parasport. At baseline (*i.e*., pre-CMCL seminar), a large percentage of participants reported working with patients with physical disabilities ‘all the time’ (53.6%). When working with these patients, most participants reported discussing LTPA ‘frequently’ (29.9%), but ‘never’ discussing parasport (40.2%). Complete demographic characteristics for participants are presented in Table 1.

**Table 1:** Healthcare professionals’ demographic characteristics

**Characteristic**	**HCPs in study N = 97**
Gender	
Male	30 (30.9)
Female	65 (69.0)
Ethnicity (Caucasian)	95 (97.9)
Age (years)	36.23 ± 10.42
LTPA (days/week)	4.35 ± 1.71
Involved in parasport	3 (3.1)
Seminars delivered per province (#)	
British Columbia (2)	16 (16.5)
Saskatchewan (4)	15 (15.5)
Newfoundland/Labrador (3)	27 (27.8)
New Brunswick (4)	31 (32.0)
Prince Edward Island (1)	5 (5.2)
Type of HCP	
Physician	11 (11.3)
Physical therapist	27 (27.8)
Occupational therapist	8 (8.2)
Recreational therapist	2 (2.1)
Nurse	3 (3.1)
Educator	14 (14.4)
Other	30 (30.9)
Years in profession (years)	11.56 ± 9.94
Frequency of working with patients^a^	
Never	2 (2.1)
Rarely	10 (10.3)
Sometimes	16 (16.5)
Frequently	14 (14.4)
All the time	52 (53.6)
Frequency of discussing LTPA with patients^a^	
Never	4 (4.1)
Rarely	9 (9.3)
Sometimes	27 (27.3)
Frequently	29 (29.9)
All the time	22 (22.7)
Frequency of discussing parasport with patients^a^	
Never	39 (40.2)
Rarely	29 (29.9)
Sometimes	16 (16.5)
Frequently	4 (4.1)
All the time	2 (2.1)

*Note*: HCPs: Healthcare professionals; LTPA: Leisure-time physical activity.

All values are n (%) except for age, days of LTPA per week, and years in profession, which are M ± SD.

No seminars were delivered to HCPs in Alberta, Ontario, or Nova Scotia during the study period. Some participants declined to respond to certain questions. Hence, n <97 for some variables.

^a^Frequency specific to patients with physical disabilities.

### Protocol

The study protocol was approved by the McMaster Research Ethics Board. Following training, presenters delivered CMCL seminars to HCPs across Canada. Full details concerning CMCL’s development, behaviour change techniques, and causal processes targeted in the intervention are described in detail elsewhere [Tomasone et al., resubmitted]. Participants completed a demographic and professional information questionnaire. To assess CMCL effectiveness, immediately prior to and following the CMCL seminar, HCPs completed hardcopy measures assessing their social cognitions for discussing LTPA with their patients with a physical disability. To assess maintenance of change in social cognitions, participants were emailed a link to an online questionnaire at one and six months following their attendance^a^. To gather information about CMCL intervention implementation components, presenters completed a Presenter Checklist following the delivery of each seminar.

### Measures

#### Social cognitions for discussing LTPA with patients

Attitudes and subjective norms were assessed with items adapted from Ajzen [27], and PBC was assessed by items adapted from Rhodes and Courneya [28]. All social cognition items were rated on a 7-point Likert scale. For TPB construct scales with more than two items, item scores were averaged to give an overall construct score. Table 2 lists the items, response scale, and the internal reliability or correlation of the items for each scale included in the social cognition questionnaire.

**Table 2:** Questionnaire items assessing healthcare professionals’ social cognitions for discussing leisure-time physical activity with their patients

**Theory of planned behaviour construct (# items)**		**Response scale**	**Internal reliability score (α) or correlation (r)**
**Items included in scale**			
**Attitudes (5 items)**			αs ≥0.81
*Instrumental attitudes*		1 = Strongly disagree,	
1. Attending this CMCL presentation will help me discuss physical activity and parasport to my patients with a physical disability.		7 = Strongly agree	
*Affective attitudes*		Anchors represent extremes (1/7) on 7-point Likert scale	
2. Complete the statement, ‘Discussing physical activity and parasport to my patients with a physical disability would be__________’.			
	a. Harmful/beneficial		
	b. Worthless/valuable		
	c. Difficult/easy		
	d. Unpleasant/pleasant		
**Subjective norms (1 item)**		1 = Strongly disagree,	N/A
1. Other health care professionals that I work with think I should discuss physical activity and parasport with my patients with a physical disability.		7 = Strongly agree	
**Perceived behavioural control (2 items)**		1 = Not at all confident,	*r*s ≥0.44
If you were really motivated and had all the resources that you needed, how confident are you in your ability to…		7 = Completely confident	*p*s ≤0.03
1. …discuss physical activity and parasport with your patients with a physical disability?			
2. …persuade your patients with a physical disability to participate in physical activity and parasport?			
**Intentions (3 items)**		1 = Strongly disagree,	αs ≥0.82
1. In the next four weeks, I intend to seek out additional information about physical activity and parasport for my patients/clients with a physical disability.		7 = Strongly agree	
2. In the next four weeks, I intend to seek out additional information to use to persuade my patients with a physical disability to engage in physical activity and parasport.			
3. In the next four weeks, I intend to persuade my patients with a physical disability to engage in physical activity and parasport.			

*Note*. CMCL: ‘Changing Minds, Changing Lives.’ The column indicating scale internal reliability (Cronbach alpha) scores for the items on the scale and Pearson correlations between the items on the scale represent the lowest value across the four time points (pre-CMCL, post-CMCL, 1-month follow-up, 6-month follow-up). All internal reliability scores were acceptable.

### Implementation variables

Ten different implementation variables were considered for the current study: three presenter characteristics and seven intervention delivery components. Presenter characteristics data were obtained from a demographic questionnaire that interventionists completed during their CMCL training session [Tomasone et al., resubmitted]. Intervention delivery components data were obtained from Presenter Checklists which served as both a ‘roadmap’ for consistent delivery of the CMCL curriculum and as an implementation data collection tool^b^. Table 3 provides the operationalization and summary of the implementation variables. As a reliability check, the first author attended and completed a Presenter Checklist for two CMCL seminars delivered by two different presenters. For checklist items included in the current study (n = 7), agreement between the researcher and Presenters was high (86% and 100%).

**Table 3:** Operationalization and summary of the ten implementation variables considered in predicting change in social cognitions

**Implementation variables**	**Abbreviation**	**Continuous variable**	Dichotomous variable*	
		**Range**	**Yes**	**No**
*Presenter characteristics*				
Seminar number using the new CMCL curriculum	CMCL#	1–4		
Years the presenter has been part of CMCL staff	CMCLyears	0-5		
Whether the presenter is a HCP themselves	HCPpresenter		66	26
*Intervention delivery components*				
Number of attendees present	Attendees	8–77		
Duration (minutes)	Duration	60–120		
Parasport athlete present at seminar to share his/her experience with the role his/her HCP played in his/her LTPA success	Athlete		85	7
Parasport equipment available for viewing and use by attendees	Equipment		42	50
Educational resources about LTPA for people with a physical disability distributed to attendees	Resources		87	5
Inclusion of audio-visual component (e.g., photos, videos) not part of standard CMCL curriculum	AVadded		14	78
Partner with community organization	Partner		21	71

*Note*. AV: Audiovisual; CMCL: ‘Changing Minds, Changing Lives’; HCP: Healthcare professional; LTPA: Leisure-time physical activity. Data for the presenter characteristics were extracted from presenter demographic questionnaires completed prior to interventionist training. Data for the intervention delivery components were extracted from the Presenter Checklists that were completed for 14 of the 15 seminars delivered to HCPs during the study period.

*Number of participants exposed to each implementation variable over the 14 seminars for which Presenter Checklists are available. The seminar that is missing a Presenter Checklist had five participants attend; hence, frequencies in the last column add up to 92.

### Statistical analyses

#### Data handling

All data were screened for outliers and normality using established guidelines [29]. Complete TPB questionnaire data were available for 96%, 91%, 26% and 40% of the 97 HCPs at pre, post, one, and six months, respectively. Missing value analysis indicated that data were missing at random for all demographics and TPB variables at all four time points except for HCPs’ profession length. Specifically, the six HCPs who did not provide their profession length had lower intentions post-CMCL than the HCPs who provided the number of years they had been practicing. Since this variable was not missing at random, and there was a large amount of missing data at one and six months (74% and 60%, respectively), missing data were imputed using multiple imputation methods prior to conducting analyses. Multiple imputation is an appropriate and respected method for dealing with large amounts of missing data because it can be used to incorporate auxiliary information about the missing data into the final analysis (*e.g*., profession length [30]), and it gives standard errors and p-values that incorporate missing data uncertainty [31]. In total, 10 imputation data sets were created, as recommended by Rubin [32], and subsequent analyses were conducted separately on each dataset; however, for simplicity of reporting, only pooled results are included in the Results section^c^.

### Changes in social cognitions over time

Separate repeated measures ANOVAs were performed for each of the four TPB cognitions over the four time points. Significant univariate effects were followed up by paired sample t-tests to identify significant changes in the TPB variables between pre-post, pre-1 month, pre-6 month, post-1 month, post-6 month, and 1–6 month. Alpha was adjusted using the Bonferroni method for the multiple comparisons (α = 0.05/6 comparisons per TPB cognition = 0.0083). Cohen’s d was calculated as an index of effect size.

### Using the TPB to predict intentions to discuss LTPA

In accordance with Ajzen’s TPB [17], a hierarchical regression analysis was conducted to predict intentions with attitudes and subjective norms entered in the first block, and PBC entered in the second block. A TPB regression predicting intentions was conducted for any time point at which any implementation variables emerged as significant predictors of the TPB constructs.

### Using implementation variables to predict change in social cognitions

Residualized change scores were calculated for each TPB cognition at any time point that was significantly different from its pre-seminar value. Continuous implementation variables that were significantly correlated with a change score were included as predictors of that TPB cognition in subsequent regression models. Following independent t-tests, categorical implementation variables whose presence resulted in a significant difference in a change score were included as predictors of that TPB cognition in subsequent regression models.

Hierarchical multiple regressions were performed to predict each TPB cognition at time points that significantly differed from baseline. The pre-seminar value of the cognition was entered first, presenter characteristics were entered second, and intervention delivery components were entered last. Implementation variables were entered into their own block so that changes in R^2^ values could be calculated for each variable.

## Results

### Changes in social cognitions over time

Pooled descriptive statistics for the TPB variables at each time point are presented in Table 4. Mauchly’s Test of Sphericity indicated that the assumption of sphericity had been violated for all TPB variables; thus, a Greenhouse-Geisser correction was used. There was a significant effect of time on all TPB variables (see Table 4 for ANOVA results). Despite initially high values for each social cognition (all Ms ≥4.35 out of 7), follow-up paired samples t-tests revealed significant increases in attitudes, subjective norms, PBC, and intentions to discuss LTPA and parasport from pre- to post-CMCL seminar (all ps ≤0.002). Significant decreases in all four TPB cognitions were seen between post-CMCL and 6-month follow-up (all ps ≤0.005); however, these follow-up values were not significantly different from baseline (p ≥0.14). Significant decreases in attitudes and intentions to discuss LTPA and parasport with patients with a physical disability were also seen between post-CMCL and 1-month follow-up (ps ≤0.006). See Table 4 for paired samples t-test results.

**Table 4:** Changes in healthcare professionals’ social cognitions over time

	**Descriptive statistics**				**Repeated-measures ANOVA**	**Paired samples *t*-tests**					
	**Time**					**Cohen’s *d***					
**Construct**	**Pre**	**Post**	**1-month**	**6-month**	***F*-value**	**Pre-post**	**Pre-1mo**	**Pre-6mo**	**Post-1mo**	**Post-6mo**	**1mo-6mo**
Attitudes	5.84 ± 0.86	6.32 ± 0.07	5.41 ± 0.18	5.58 ± 0.17	33.7***	0.76**	-0.54	-0.25	-0.95**	-0.80**	0.15
Subjective norms	4.70 ± 0.19	5.30 ± 0.16	5.02 ± 0.24	4.53 ± 0.22	7.57**	0.35**	0.15	-0.09	-0.14	-0.44**	-0.33
PBC	4.61 ± 0.14	5.77 ± 0.09	5.45 ± 0.28	4.74 ± 0.21	34.98***	1.15**	0.61*	0.08	-0.28	-0.90**	-0.47
Intentions	4.35 ± 0.13	5.31 ± 0.14	4.51 ± 0.27	3.95 ± 0.25	25.79***	1.00**	0.11	-0.22	-0.53**	-0.80**	-0.30

*Note*. PBC: Perceived behavioural control. Descriptive statistics (*M* ± *SE*) and repeated-measures ANOVA *F*-values and *p*-values are pooled across the 10 multiple imputation data sets.

*indicates test value reached statistical significance at *p* ≤ 0.05.

**indicates test value reached statistical significance at *p* ≤ 0.0083 (Bonferroni correction for multiple comparisons used for *t*-tests).

***indicates test value reached statistical significance at *p*. < 001.

### Using the TPB to predict intentions to discuss LTPA post-seminar

The results of the hierarchical regression analysis predicting intentions are presented in Table 5. In the first step, both post-seminar attitudes (β = 0.27, p <0.05) and subjective norms (β = 0.38, p <0.001) were significant predictors of intentions, accounting for 31% of the variance (AdjR^2^ = 0.30). In the second step, post-seminar PBC accounted for an additional 10% of explained variance and was a significant predictor (β = 0.45, p <0.001). Subjective norms remained a significant predictor (β = 0.34, p <0.001) but attitudes did not (β = -0.03, p >0.05). The overall regression model was significant, accounting for 41% of the variance in post-seminar intentions (AdjR^2^ = 0.39, F(1, 93) = 15.18, p <0.001).

**Table 5:** Hierarchical multiple regression analysis predicting healthcare professionals’ intentions immediately following the CMCL-seminar

**Steps/predictors**	***R***^***2***^	**Adj** ***R***^***2***^	***R***^***2 ***^**change**	***F *** **change**	***df***	**β**_**1**_	**β**_**2**_
1. Attitudes						0.27*	-0.03
Subjective norms	0.31	0.30		21.64**	2,94	0.38**	0.34**
2. PBC	0.41	0.39	0.10	15.18**	1,93		0.45**

*Note.* PBC: Perceived behavioural control.

*p <0.05; **p ≤0.01. β_1_ represents the standardized beta coefficients for regression Equation 1. β_2_ represents the standardized beta coefficients for regression Equation 2.

### Using implementation variables to predict change in social cognitions

From pre- to post-seminar, participants had a significantly lower attitude change score if they attended a seminar where an audiovisual component was added (‘AVadded’; t(90) = 2.18, p = 0.03). Longer seminars (‘duration’) were negatively associated with attitude change (r = -0.24, p = 0.02) and PBC (r = -0.23, p = 0.03). The number of seminars that the presenter had delivered using the new curriculum (‘CMCL#’) was also negatively correlated with PBC (r = -0.23, p = 0.03). No other correlations were significant from pre- to post-CMCL seminar. No implementation variables emerged as being significantly related to a change in TPB cognitions from post-seminar to 1- or 6-month follow-up.

### Predicting change in attitudes and PBC

Significant predictors of changes in attitudes and PBC between pre- and post-seminar were then entered into separate hierarchical regression models to predict each post-seminar TPB variable. The model predicting post-seminar attitudes accounted for 39% of the variance (AdjR^2^ = 0.37); however, pre-seminar attitudes emerged as the only significant predictor (β = 0.60, p <0.001) of post-seminar attitudes. The final model predicting post-seminar PBC accounted for 40.9% of the variance (AdjR^2^ = 0.39) with both pre-seminar PBC (β = 0.58, p <0.001) and the number of seminars (‘CMCL#’; β = -0.18, p <0.05) emerging as significant predictors of participants’ perceptions of control following the seminar. Complete regression results for predicting participants’ post-seminar attitudes and PBC can be found in Tables 6 and 7, respectively.

**Table 6:** Hierarchical multiple regression analysis predicting healthcare professionals’ attitudes immediately following the CMCL seminar

**Steps/predictors**	***R***^***2***^	**Adj** ***R***^***2***^	***R***^***2 ***^**change**	***F *** **change**	***df***	**β**_**1**_	**β**_**2**_	**β**_**3**_
1. Attitudes (pre)	0.35	0.34		48.23**	1,90	0.59**	0.62**	0.60**
2. AVadded	0.38	0.37	0.03	4.64**	1,89		-0.18*	-0.14
3. Duration	0.39	0.37	0.01	6.01**	1,88			-0.22

*Note.* AV: Audiovisual.

*p <0.05; **p ≤0.01. β_1_ represents the standardized beta coefficients for regression Equation 1. β_2_ represents the standardized beta coefficients for regression Equation 2, and β_3_ represents the standardized beta coefficients for regression Equation 3.

**Table 7:** Hierarchical multiple regression analysis predicting healthcare professionals’ perceived behavioural control immediately following the CMCL seminar

**Steps/predictors**	***R***^***2***^	**Adj** ***R***^***2***^	***R***^***2 ***^**change**	***F *** **change**	***df***	**β**_**1**_	**β**_**2**_	**β**_**3**_
1. PBC (pre)	0.36	0.35		45.24**	1,90	0.60**	0.59**	0.58**
2. CMCL#	0.40	0.38	0.04	5.96**	1,89		-0.20*	-0.18*
3. Duration	0.41	0.39	0.01	1.48**	1,88			-0.10

*Note.* CMCL: ‘Changing Minds, Changing Lives’; PBC: Perceived behavioural control.

*p <0.05; **p ≤0.01. β_1_ represents the standardized beta coefficients for regression Equation 1. β_2_ represents the standardized beta coefficients for regression Equation 2, and β_3_ represents the standardized beta coefficients for regression Equation 3.

## Discussion

Following a single, seminar-mediated educational intervention, HCPs reported significant increases in their attitudes, subjective norms, PBC, and intentions to discuss LTPA with patients with physical disabilities; however, these increases in social cognitions were not maintained at 1- and 6-months follow-up. Participants’ PBC emerged as the strongest predictor of post-seminar intentions to discuss LTPA with patients with physical disabilities. Contrary to our hypotheses, intervention delivery components did not emerge as significant predictors of attitudes and PBC, and presenter characteristics did not emerge as significant predictors of subjective norms, at post-seminar. Only the number of seminars that the presenter had delivered emerged as a significant negative predictor of post-seminar PBC.

Despite HCPs’ strong attitudes, subjective norms, PBC, and intentions for LTPA prior to participating in a CMCL seminar, significant increases were seen for all four TPB cognitions following the seminar. These results are encouraging given that the 15 seminars delivered during the study period used a recently revised CMCL curriculum that is framed around the TPB [Tomasone et al., resubmitted]; hence, the curriculum appears to be effectively targeting the TPB constructs. As hypothesized, the findings from the current study suggest that CMCL is effective for increasing HCPs’ social cognitions for discussing LTPA immediately following their participation in a CMCL seminar.

In line with our hypothesis, the significant increases in participants’ social cognitions were not maintained at one and six-month follow-up. This is consistent with previous reviews demonstrating that traditional seminar-based educational interventions are not particularly effective at maintaining change in physicians’ [25] and allied healthcare professionals’ [33] behaviour over time. In the current study, the theoretical determinants of behaviour were not sustained at either follow-up point, suggesting that a single CMCL seminar is not sufficient for short- or long-term maintenance of social cognitions. The CMCL intervention may benefit from the inclusion of additional knowledge translation strategies (*e.g*., audit and feedback, point of decision prompts, email reminders; [26]) or ‘booster’ sessions to preserve HCPs’ strong social cognitions for discussing LTPA over time.

Post-seminar attitudes, subjective norms, and PBC accounted for 40.9% of the variance in HCPs’ intentions. In contrast, previous research has shown that the TPB constructs can only account for 11.6% to 30.0% of the variance in intentions to engage in various clinical behaviours [19]. Our results speak to the relevance of the TPB for explaining intentions to discuss LTPA in clinical settings. PBC emerged as the social cognition with the largest impact on intentions. This finding is of interest, as post-seminar PBC was the only cognition to be predicted by an implementation variable. The assessment of barriers and facilitators to discussing LTPA in day-to-day practice would provide insight into how the CMCL intervention can be modified to maintain HCPs’ PBC following the CMCL seminar.

While several presenter characteristics and intervention delivery components were related to changes in social cognitions between pre- and post-seminar, the only implementation variable that emerged as a significant predictor of any social cognition was the number of seminars that a presenter had delivered. This finding seems counter-intuitive since it would be expected that presenter experience with the new curriculum would lead to higher quality seminar delivery and, thus, be positively related with participants’ outcomes. However, the CMCL presenters were trained to use the new CMCL curriculum in a single session, prior to their first delivery of a CMCL seminar [Tomasone et al., resubmitted]. Following training, on-going consultation and problem-solving with the presenters may have improved implementation over time, as demonstrated in previous research [23]. It is possible that experienced presenters ventured further away from the set curriculum and, therefore, did not adequately deliver the material designed to target the TPB constructs. Also, as previously reported [Tomasone et al., resubmitted], presenters reported a decrease in PBC for delivering the curriculum over the study period. While this decrease in PBC may be an indication of a curriculum reinvention process [34], it could also indicate a waning of presenters’ confidence for delivering the new curriculum with increasing seminar delivery number. For example, Durlak and DuPre have identified providers’ self-efficacy (similar to PBC) for delivering an intervention as a factor influencing implementation and, hence, subsequent outcomes among intervention recipients [23]. Future research should examine presenters’ PBC immediately prior to each CMCL seminar in order to assess the impact of providers’ PBC on attendees’ PBC.

Adding an audiovisual component to the CMCL intervention was negatively related to, and a significant predictor of, post-seminar attitudes when pre-seminar attitudes was the only variable included in the regression model. During the participatory curriculum development process [Tomasone et al., resubmitted], the CMCL curriculum underwent a thorough review process and now includes evidence-based best-practices for behaviour change interventions [35]. The inclusion of additional delivery components, such as images or videos, may detract from the message or flow of the curriculum, cause the presenter to rush through certain theory-based components in order to accommodate the added component, and/or increase the seminar duration. Indeed, seminar duration was a negative correlate of both attitudes and PBC post-seminar. A common barrier reported by HCPs is a perceived lack of time during their work day [36]; thus, HCPs may feel annoyed or get anxious to leave a lengthy educational seminar, feelings that could undermine the impact of the seminar. The fact that duration was not a significant predictor of either cognition suggests that it may not necessarily be duration alone that impacts cognitive outcomes, but how the seminar time is used. For example, a presenter may stumble through slides or lose focus, increasing seminar duration while also leading attendees to feel like their limited time is being wasted. Presenter skill and proficiency for seminar delivery was not examined in the current study but is an interesting avenue for future research that uses implementation variables to predict intervention outcomes.

### Study strengths and limitations

Previous research examining the impact of implementation on intervention outcomes has been correlational or comparative (*e.g*., comparing those who received intervention outcomes vs. those who did not [23]). To our knowledge, this is one of the first studies to use implementation variables to predict intervention outcomes to help generate an understanding of the mechanisms by which HCPs’ social cognitions change. In their review, Durlak and DuPre report that there are at least 23 factors that might affect implementation [23]; therefore, examining the variables that enhance an intervention’s effectiveness is important so that an intervention can continue to make an impact as its reach increases, and so that key implementation components are included in future, more cost-efficient iterations of the intervention [22]. Further, examining implementation variables alongside the TPB social cognitions has provided a generalizable framework for future exploration of the causal mechanisms of change in HCPs’ cognitions [18,25,37]. The theoretically-informed approach used in the current study can be adopted by other researchers and knowledge translation practitioners in the development, dissemination and evaluation of nationwide initiatives aimed at educating HCPs.

Despite these strengths, there are some limitations to the current study. First, the low response rate and potential self-selection bias may influence the study’s internal validity. The participants in the current study represent only 30% of the HCPs who attended the 15 CMCL seminars during the study period. However, Eccles and colleagues reported a 21 to 48% response rate on theory-based questionnaires over a series of five studies examining clinical behaviours among HCPs [19], suggesting that the response in the current study is typical in studies of HCPs. In addition, the HCPs opted into participating, introducing a potential self-selection bias. The participants were an active sample (reported engaging in LTPA on at least four days of the week, on average) and reported discussing LTPA with their patients with a physical disability at least ‘sometimes', if not ‘frequently’ or ‘all the time'. The representation of non-participating HCPs was not measured, so there is no way to discern whether these findings extend to all CMCL attendees. Nevertheless, findings from Bower and colleagues indicate that HCPs who participate in continuing education select the opportunities that appeal to them [38], suggesting that all HCPs’ who attended the CMCL seminars may already value a physically active lifestyle for both themselves and their patients.

Another limitation is that prescription behaviour (*i.e*., discussion of LTPA) was not assessed in the current study due to the logistical feasibility of nationwide monitoring, as well as patient privacy and confidentiality concerns [37]. However, a large effect size for change in intentions from pre- to post-seminar was seen (d = 1.00). A review of studies examining the relationship between intentions and clinical behaviour shows that intentions accounts for 15% to 40% of the variance in HCP behaviour, making intention a reasonable proxy measure for behaviour change in theory-based interventions aimed at HCPs [37].

## Conclusions

The theory- and evidence-based seminars were effective at increasing HCPs’ social cognitions for discussing LTPA with their patients with physical disabilities immediately following the seminar, but not at the 1- and 6-month follow-ups. The TPB cognitions were able to account for 40.9% of the variance in HCPs’ intentions to discuss LTPA. While the number of seminars delivered by the presenter, as well as adding an audiovisual component and increasing seminar duration, were negatively related to changes in cognitions from pre- to post-seminar, the only implementation variable that emerged as a predictor of cognitions (PBC in particular) was the number of seminars that the presenter had delivered. It is suggested that subsequent iterations of the CMCL intervention include additional strategies to sustain improvements in HCPs’ social cognitions over time. Future evaluations of the CMCL intervention should measure additional implementation variables, such as presenter self-efficacy at time of presentation and proficiency of delivery, so that the key ingredients for ‘Changing Minds’ can continue to be investigated.

## Endnotes

^a^Three email attempts were made for each participant at 1-month follow-up. Due to the low response rate at this time point (n = 25, 26%), the protocol was adjusted at 6-months follow-up so that three email attempts were followed by three telephone attempts to contact the HCPs. This increased the response rate of the 6-month questionnaire to 40% (n = 39).

^b^Presenter Checklists were completed for 14 of the 15 seminars delivered during the study period. Note that both the presenter demographic questionnaire and Presenter Checklists are available from the first author.

^c^Pooled results were obtained by averaging the corresponding F- and t*-*values, regression coefficients, and R^2^ values that were not pooled by SPSS [32,39]. Standard errors were pooled using the equations outlined in Baraldi and Enders [39]. These pooled standard errors were used to determine the significance of the pooled F-tests, t-tests, and regression models.

## Abbreviations

CMCL: ‘Changing Minds, Changing Lives’; HCPs: Healthcare professionals; LTPA: Leisure-time physical activity; PBC: Perceived behavioural control; TPB: Theory of Planned Behaviour.

## Competing interests

JRT and KMG sat on the ‘Changing Minds, Changing Lives’ Advisory Committee when the intervention curriculum was being restructured. LD was the Manager of Sport Development at the Canadian Paralympic Committee during the study period. PAE has no competing interests to report.

## Authors’ contributions

JRT conceived of the study, participated in its design and execution, and was responsible for data collection, synthesis and analyses. KMG, PAE, and LD participated in the study’s design and coordination. JRT and KMG were directly involved in the preparation of this manuscript. All authors read and approved the final manuscript prior to submission.
